# Supplementary material for: ZFYVE28 mediates insulin resistance by promoting phosphorylated insulin receptor degradation via increasing late endosomes production
Source: Nat Commun. 2023 Oct 26;14:6833. doi: 10.1038/s41467-023-42657-w (PMC10603069; doi:10.1038/s41467-023-42657-w)
Supplement: Supplementary file 1 — Supplementary Information [file 41467_2023_42657_MOESM1_ESM.pdf]

Supplementary information

Title: ZFYVE28 mediates insulin resistance by promoting phosphorylated insulin receptor degradation via increasing late endosomes production

Liang Yu[1, 3], Mengchen Xu[1, 3], Yupeng Yan[1, 3], Shuchen Huang[1, 3], Mengmeng Yuan[1], Bing Cui[1], Cheng Lv[1], Yu Zhang[1], Hongrui Wang[1], Xiaolei Jin[2], Rutai Hui[1], Yibo Wang[1]*

1, State Key Laboratory of Cardiovascular Disease, Fuwai Hospital, National Center for Cardiovascular Diseases, Chinese Academy of Medical Sciences and Peking Union Medical College, Beijing, China.

2, Plastic Surgery Hospital, Chinese Academy of Medical Sciences & Peking Union Medical College, Beijing, China.

3, these authors contributed equally: Liang Yu, Mengchen Xu, Yupeng Yan, Shuchen Huang.

***Correspondence:** Yibo Wang, State Key Laboratory of Cardiovascular Disease, Fuwai Hospital, National Center for Cardiovascular Diseases, Chinese Academy of Medical Sciences and Peking Union Medical College, 167 Beilishi Rd, Beijing, 100037, China. E-mail: yibowang@hotmail.com.

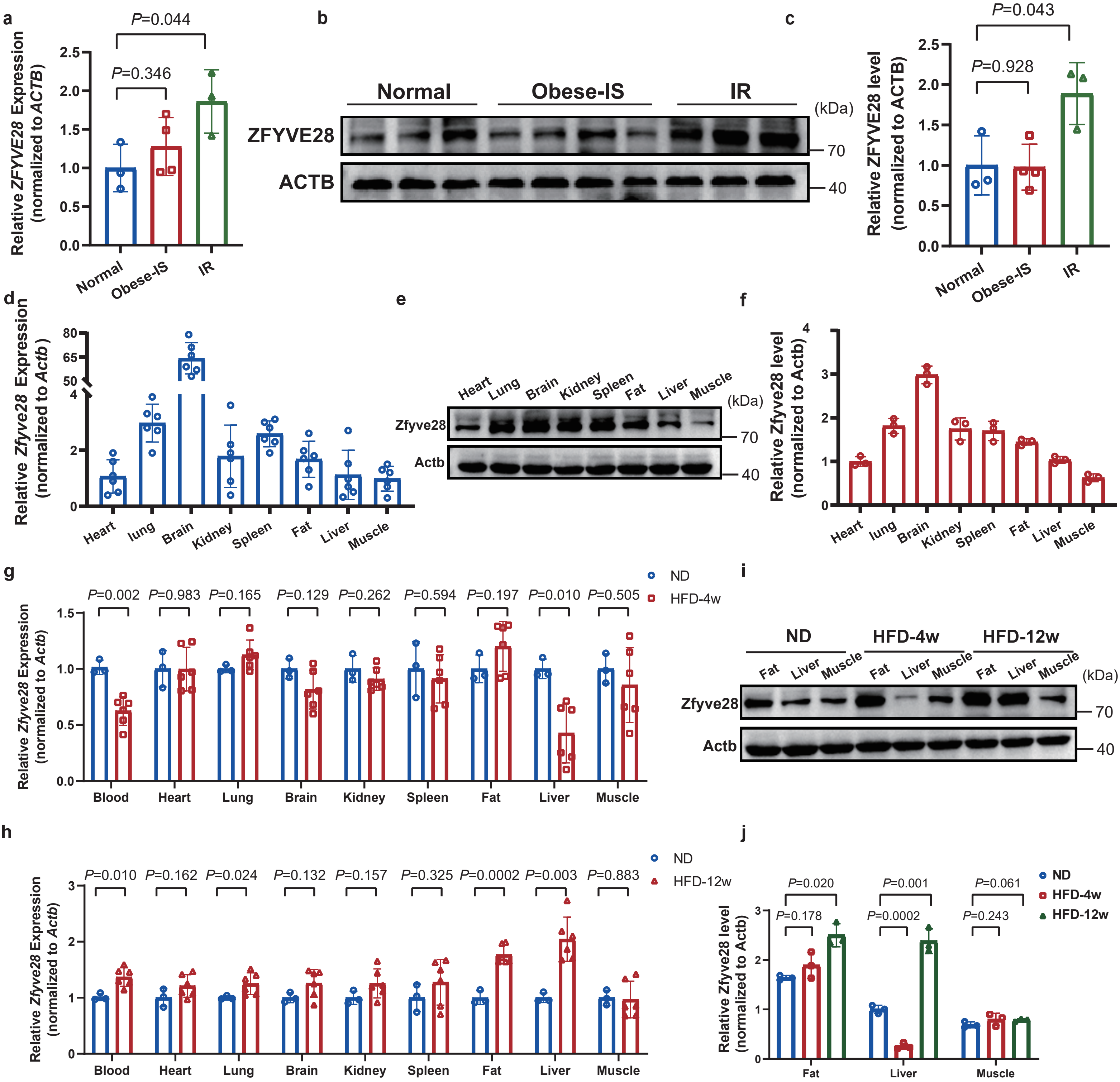

a
P=0.044
P=0.346
Relative ZFYVE28 Expression (normalized to ACTB)
Normal
Obese-IS
IR
b
Normal
Obese-IS
IR
ZFYVE28
ACTB
(kDa)
70
40
c
P=0.043
P=0.928
Relative ZFYVE28 level (normalized to ACTB)
Normal
Obese-IS
IR
d
Relative Zfyve28 Expression (normalized to Actb)
Heart
lung
Brain
Kidney
Spleen
Fat
Liver
Muscle
e
Heart
Lung
Brain
Kidney
Spleen
Fat
Liver
Muscle
Zfyve28
Actb
(kDa)
70
40
f
Relative Zfyve28 level (normalized to Actb)
Heart
lung
Brain
Kidney
Spleen
Fat
Liver
Muscle
g
ND
HFD-4w
P=0.002 P=0.983 P=0.165 P=0.129 P=0.262 P=0.594 P=0.197 P=0.010 P=0.505
Relative Zfyve28 Expression (normalized to Actb)
Blood
Heart
Lung
Brain
Kidney
Spleen
Fat
Liver
Muscle
i
ND
HFD-4w
HFD-12w
Fat
Liver
Muscle
Zfyve28
Actb
(kDa)
70
40
h
ND
HFD-12w
P=0.010 P=0.162 P=0.024 P=0.132 P=0.157 P=0.325 P=0.0002 P=0.003 P=0.883
Relative Zfyve28 Expression (normalized to Actb)
Blood
Heart
Lung
Brain
Kidney
Spleen
Fat
Liver
Muscle
j
ND
HFD-4w
HFD-12w
P=0.020
P=0.178
P=0.001
P=0.0002
P=0.061
P=0.243
Relative Zfyve28 level (normalized to Actb)
Fat
Liver
Muscle

Supplementary Fig. 1 Expression patterns of Zfyve8 in different tissues
a-c The mRNA (a) and protein (b) expression levels of ZFYVE28 in human fat tissues (3 control samples, 4 insulin-sensitive obese samples, 3 insulin-resistant samples). The quantitative analysis results of western blots (c) are presented. **d-f** Zfyve28 mRNA (n=6 biologically independent samples) and protein (n=3 biologically independent samples) expression levels in different tissues of WT mice. Representative western blots (e) and quantitative analysis results of three independent experiments (f) are shown. **g-h** Relative mRNA expression levels of *Zfyve28* in different samples of mice in the ND group, HFD-4w group and HFD-12w group. ND: n=3; HFD-4w: n=6; HFD-12w: n=6 biologically independent mice. **i-j** Representative western blots of Zfyve28 in the fat, liver, and skeletal muscle of mice in the ND group, HFD-4w group and HFD-12w group (i). The quantitative analysis results of three independent experiments are presented (j). Data are shown as means ± SD, and *P* values are determined by unpaired two-tailed Student's *t*-test (a, c, g-h, j). The exact *P* values are shown in the figure. Source data are provided as a Source Data file.

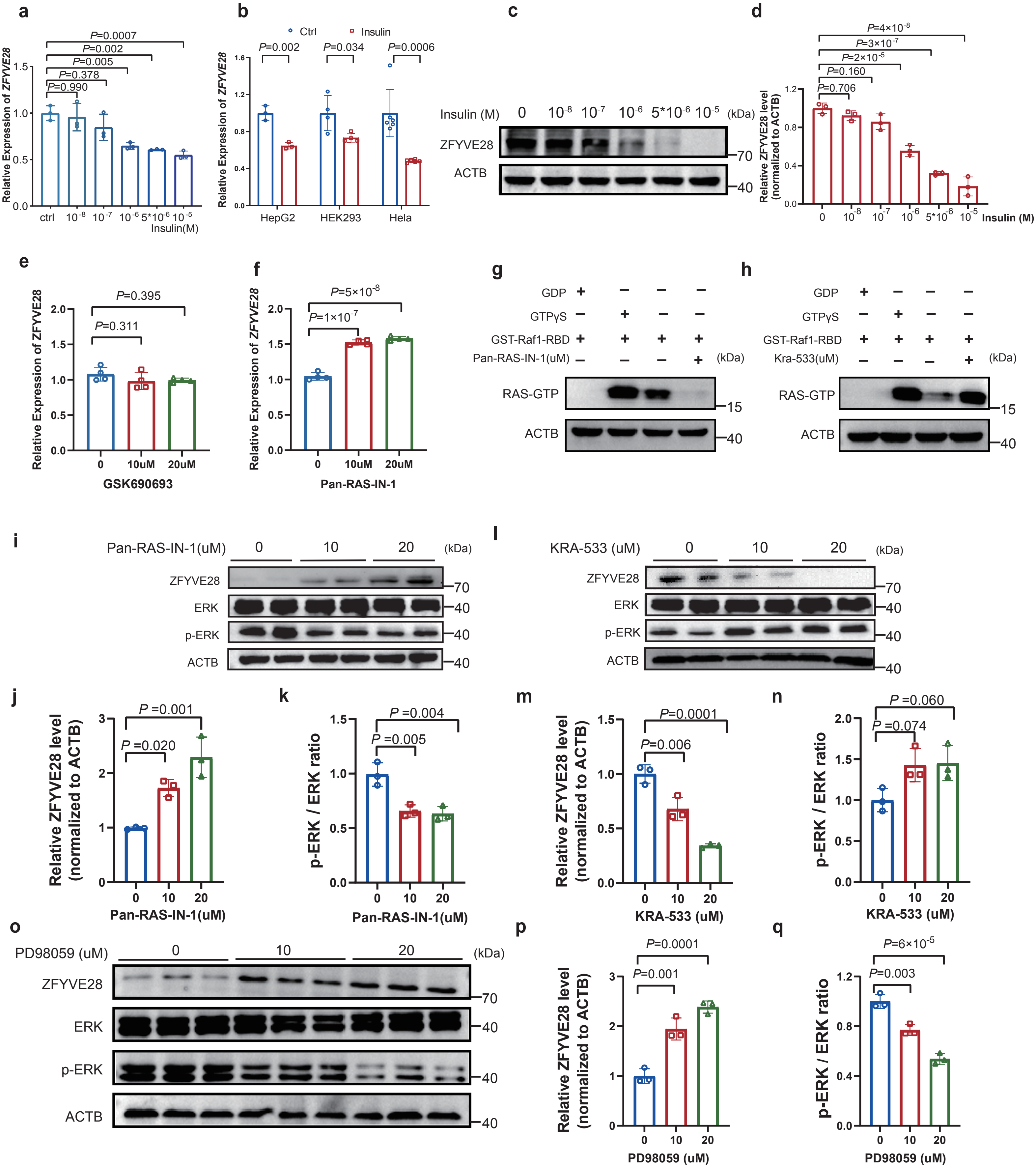
a
b
c
d
e
f
g
h
i
l
j
k
m
n
o
p
q
Relative Expression of ZFYVE28
Ctrl
Insulin
HepG2
HEK293
Hela
Insulin (M)
ZFYVE28
ACTB
(kDa)
Relative ZFYVE28 level (normalized to ACTB)
GSK690693
Pan-RAS-IN-1
GDP
GTPγS
GST-Raf1-RBD
Pan-RAS-IN-1(uM)
Kra-533(uM)
RAS-GTP
ERK
p-ERK
KRA-533 (uM)
p-ERK / ERK ratio
PD98059 (uM)

Supplementary Fig. 2 ZFYVE28 expression was inhibited by insulin via the RAS/ERK pathway.

a Relative mRNA expression of *ZFYVE28* in HepG2 cells treated with different concentrations of insulin; n=3 biologically independent samples per group. **b** Relative mRNA expression of *ZFYVE28* in HepG2, HEK293T and HeLa cells treated with insulin at 10^{-6} M. HepG2: n=3; HEK293T: n=4; HeLa: n=6 biologically independent samples. **c-d** Representative western blots of ZFYVE28 in HepG2 cells treated with different concentrations of insulin. The quantitative analysis results of three independent experiments are presented**.** **e-f** Relative mRNA expression of *ZFYVE28* in HepG2 cells treated with the AKT inhibitor GSK690693 (e) and the RAS inhibitor Pan-RAS-IN-1 (f); n=4 biologically independent samples per group. **g-h** Active RAS (RAS-GTP) assay results of HepG2 cells treated with Pan-RAS-IN-1 (g) and KRA-533 (h). Independent experiments were repeated three times in parallel, and representative western blot results are shown. GDP and GTPγS were negative and positive control samples, respectively. **i-q** Representative western blot results, as well as the quantitative analysis results (n=3 biologically independent samples per group) of HepG2 cells treated with the RAS inhibitor Pan-RAS-IN-1 (i-k), RAS agonist KRA-533 (l-n) and MEK inhibitor PD98059 (o-q). Data are shown as means ± SD, and *P* values are determined by unpaired two-tailed Student's *t*-test (b) or one-way ANOVA followed by Tukey's post hoc tests (a, d-f, j-k, m-n, p-q). The exact *P* values are shown in the figure. Source data are provided as a Source Data file.

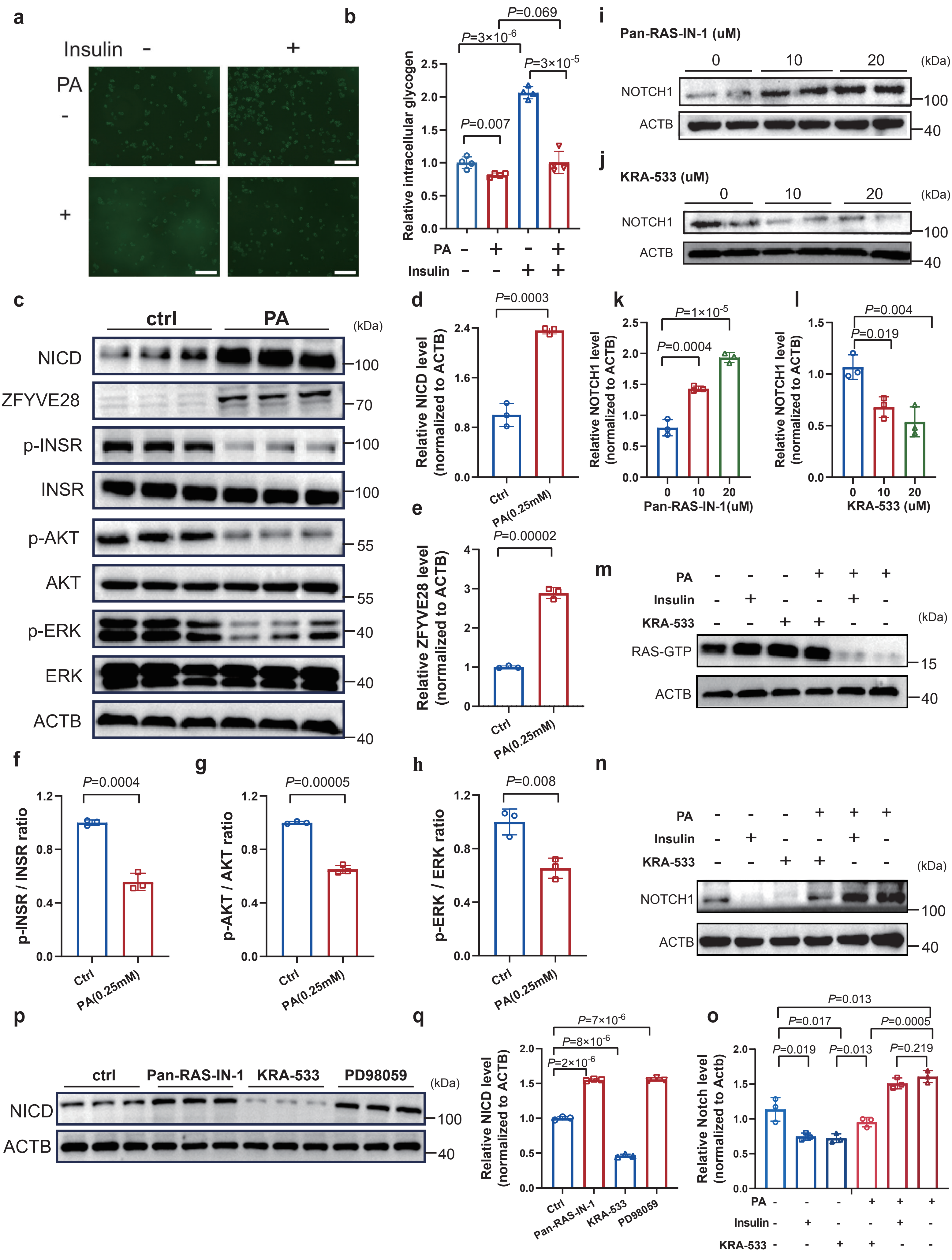
a
Insulin - +
PA - +
b
Relative intracellular glycogen
P=3×10-6
P=0.069
P=3×10-5
P=0.007
PA - + - +
Insulin - - + +
i
Pan-RAS-IN-1 (uM)
0 10 20
(kDa)
NOTCH1
ACTB
100
40
j
KRA-533 (uM)
0 10 20
(kDa)
NOTCH1
ACTB
100
40
c
ctrl PA
(kDa)
NICD
ZFYVE28
p-INSR
INSR
p-AKT
AKT
p-ERK
ERK
ACTB
100
70
100
100
55
55
40
40
40
d
P=0.0003
Relative NICD level (normalized to ACTB)
Ctrl
PA(0.25mM)
k
P=1×10-5
P=0.0004
Relative NOTCH1 level (normalized to ACTB)
Pan-RAS-IN-1(uM)
l
P=0.004
P=0.019
Relative NOTCH1 level (normalized to ACTB)
KRA-533 (uM)
e
P=0.00002
Relative ZFYVE28 level (normalized to ACTB)
Ctrl
PA(0.25mM)
m
PA - - - + + +
Insulin - + - - + -
KRA-533 - - + + - -
(kDa)
RAS-GTP
ACTB
15
40
f
P=0.0004
p-INSR / INSR ratio
Ctrl
PA(0.25mM)
g
P=0.00005
p-AKT / AKT ratio
Ctrl
PA(0.25mM)
h
P=0.008
p-ERK / ERK ratio
Ctrl
PA(0.25mM)
n
PA - - - + + +
Insulin - + - - + -
KRA-533 - - + + - -
(kDa)
NOTCH1
ACTB
100
40
p
ctrl Pan-RAS-IN-1 KRA-533 PD98059
(kDa)
NICD
ACTB
100
40
q
P=7×10-6
P=8×10-6
P=2×10-6
Relative NICD level (normalized to ACTB)
Ctrl
Pan-RAS-IN-1
KRA-533
PD98059
o
P=0.013
P=0.017
P=0.0005
P=0.019
P=0.013
P=0.219
Relative Notch level (normalized to Actb)
PA - - - + + +
Insulin - + - - + -
KRA-533 - - + + - -

Supplementary Fig. 3 NICD levels were elevated with the RAS/ERK pathway inhibited.

a-b Representative glucose uptake assay results of HepG2 cells treated with PA and insulin (a), as well as the quantitative analysis results of four independent experiments (b). Scale bar, 200 μm. **c-h** Western blot assay results of HepG2 cells treated with PA (c), as well as quantitative analysis results (d-h); n=3 biologically independent samples per group. **i-l** Representative western blots of NOTCH1 in HepG2 cells treated with Pan-RAS-IN-1 (i) and KRA-533 (j). The quantitative analysis results (k-l, n=3 biologically independent samples per group) are shown. **m** Active RAS (RAS-GTP) assay results of HepG2 cells treated with PA, insulin, and KRA-533. Independent experiments were repeated three times in parallel, and representative western blot results are shown. **n-o** Representative western blots of NOTCH1 in HepG2 cells treated with PA, insulin and KRA-533 (n), as well as the quantitative analysis results of three independent experiments (o). **p-q** Western blot assay results of NICD in HepG2 cells treated with Pan-RAS-IN-1, KRA-533 and PD98059 (p), as well as the quantitative analysis results (q); n=3 biologically independent samples per group. Data are shown as means ± SD, and *P* values are determined by unpaired two-tailed Student's *t*-test (b, d-h, o, q) or one-way ANOVA followed by Tukey's post hoc tests (k-l). The exact *P* values are shown in the figure. Source data are provided as a Source Data file.

a

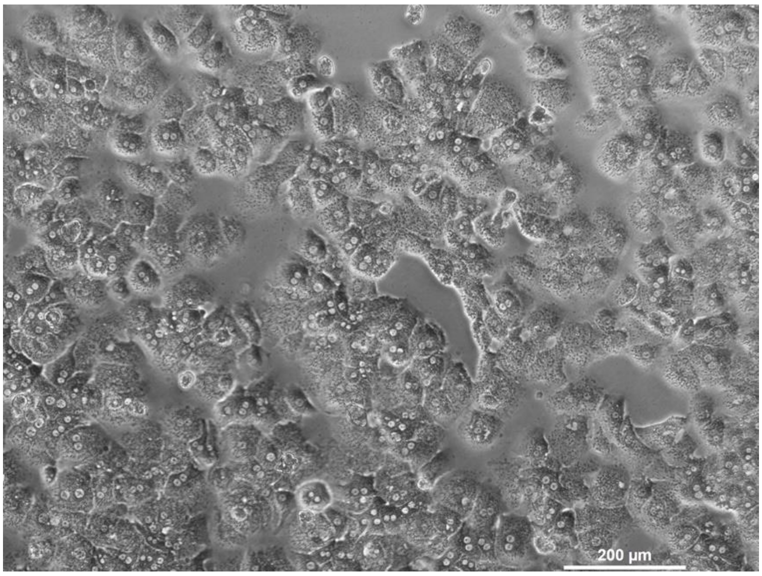

b

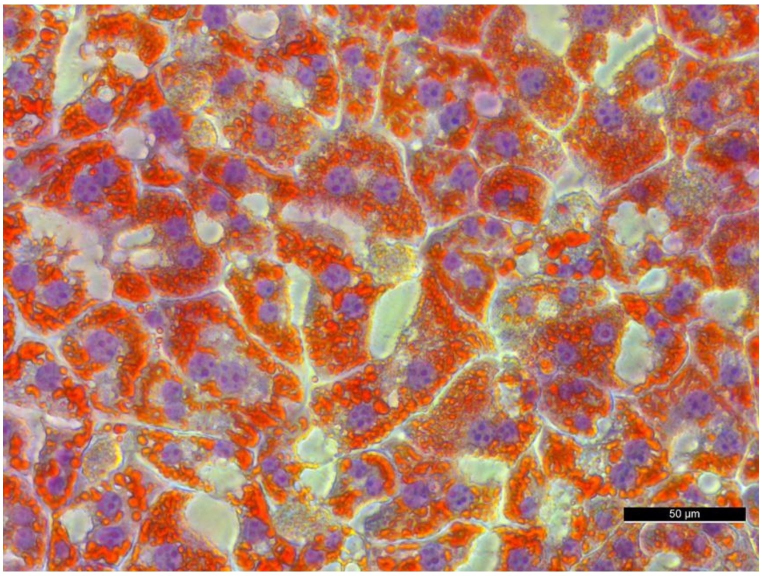

Supplementary Fig. 4 Representative image (a) and Oil Red O staining result (b) of primary hepatocytes. Scale bar, 200 µm (a), 50 µm (b).

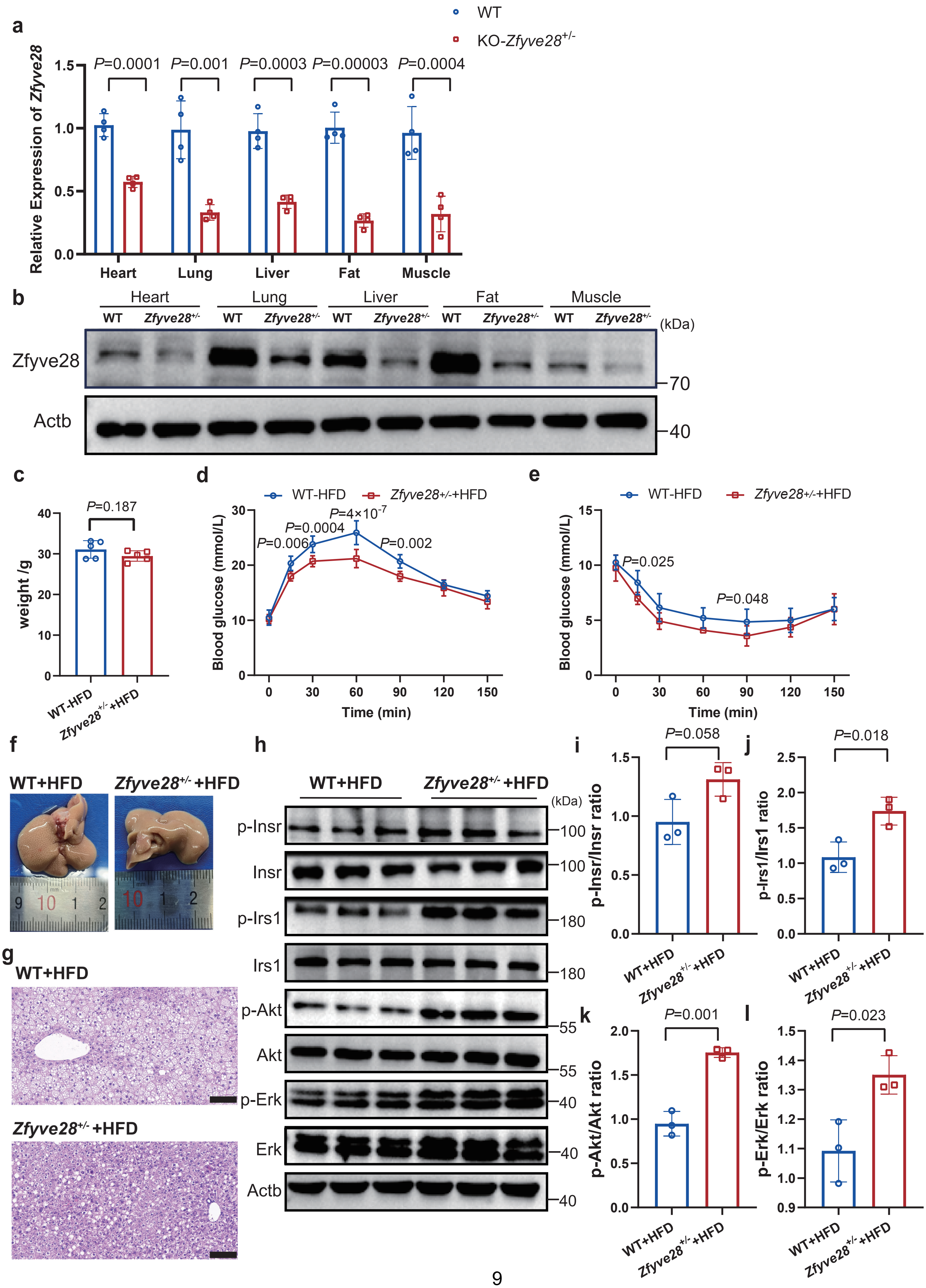
WT
KO-Zfyve28+/-
a
Relative Expression of Zfyve28
P=0.0001 P=0.001 P=0.0003 P=0.00003 P=0.0004
Heart Lung Liver Fat Muscle
b
Heart Lung Liver Fat Muscle
WT Zfyve28+/- WT Zfyve28+/- WT Zfyve28+/- WT Zfyve28+/- WT Zfyve28+/-
(kDa)
Zfyve28
Actb
70
40
c
P=0.187
weight /g
WT-HFD
Zfyve28+/- +HFD
d
WT-HFD
Zfyve28+/-+HFD
P=0.006
P=0.0004
P=4×10-7
P=0.002
Blood glucose (mmol/L)
Time (min)
e
WT-HFD
Zfyve28+/-+HFD
P=0.025
P=0.048
Blood glucose (mmol/L)
Time (min)
f
WT+HFD
Zfyve28+/- +HFD
g
WT+HFD
Zfyve28+/- +HFD
h
WT+HFD
Zfyve28+/- +HFD
(kDa)
p-Insr
Insr
p-Irs1
Irs1
p-Akt
Akt
p-Erk
Erk
Actb
100
100
180
180
55
55
40
40
40
i
P=0.058
p-Insr/Insr ratio
j
P=0.018
p-Irs1/Irs1 ratio
k
P=0.001
p-Akt/Akt ratio
l
P=0.023
p-Erk/Erk ratio
WT+HFD
Zfyve28+/- +HFD

Supplementary Fig. 5 *Zfyve28* heterozygous mice showed a slight improvement in insulin resistance.

a-b Zfyve28 expression was decreased in *Zfyve28* heterozygous mice; n=4 biologically independent samples per group (a). Representative western blot results of three independent parallel experiments are shown (b). **c** There was no difference in body weight between *Zfyve28* heterozygous mice and control mice; n=5 biologically independent mice per group. **d-e** GTT and ITT showed that *Zfyve28* heterozygous knockout improved glucose tolerance (d) and insulin sensitivity (e); n=5 biologically independent mice per group. **f-g** Representative gross images (f) and HE staining (g) of livers from *Zfyve28* heterozygous mice and control mice. Scale bar, 100 μm. **h-l** Western blot analysis (h) of hepatic protein levels in *Zfyve28* heterozygous mice and control mice. The quantification results (i-l) are shown; n=3 biologically independent samples per group. Data are shown as means ± SD, and *P* values are determined by unpaired two-tailed Student's *t*-test (a, c, i-l) or two-way ANOVA with Fisher's LSD post hoc multiple comparisons test (d-e). The exact *P* values are shown in the figure. Source data are provided as a Source Data file.

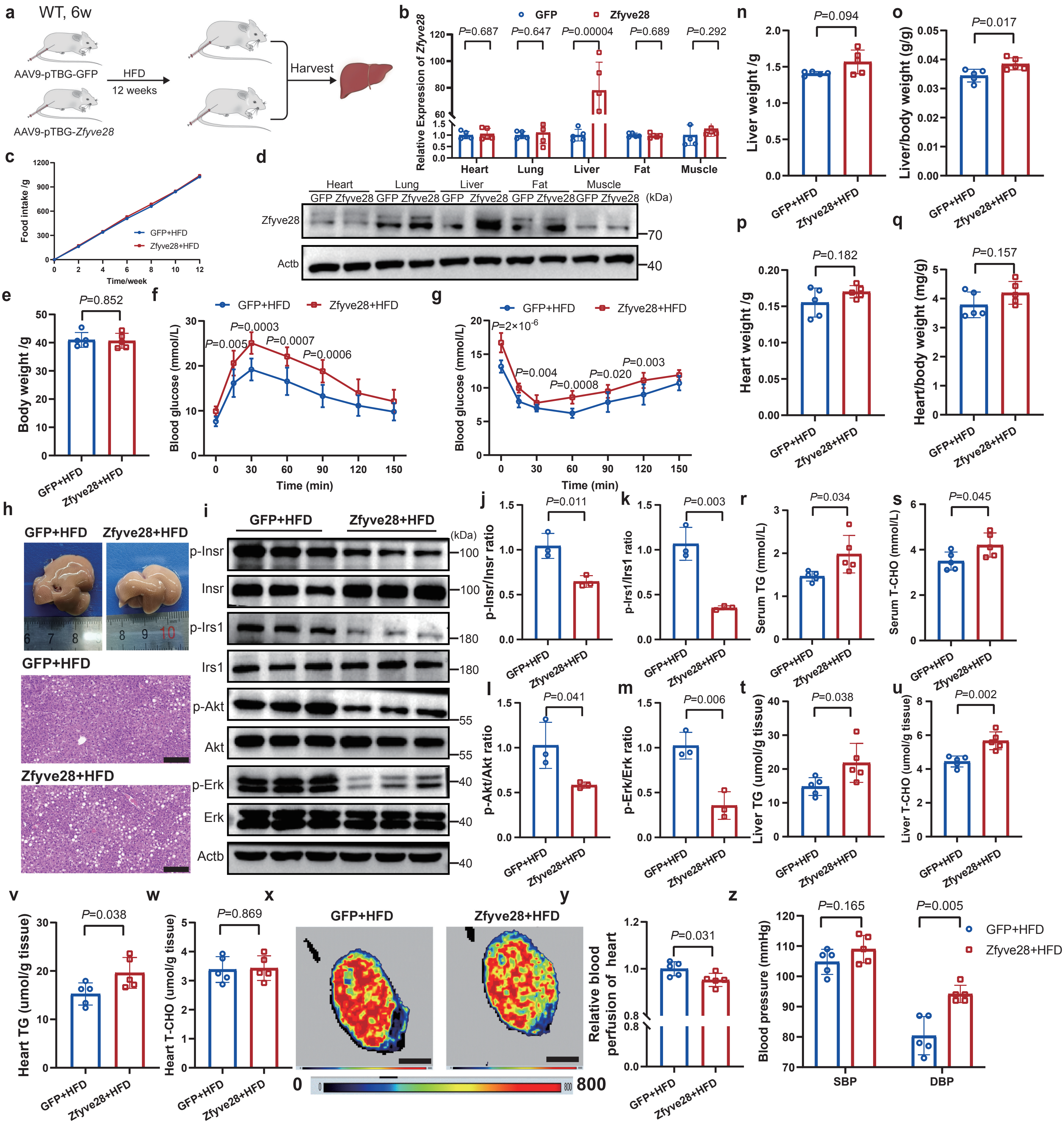

a
WT, 6w
AAV9-pTBG-GFP
AAV9-pTBG-Zfyve28
HFD
12 weeks
Harvest
b
GFP
Zfyve28
Relative Expression of Zfyve28
P=0.687
P=0.647
P=0.00004
P=0.689
P=0.292
Heart
Lung
Liver
Fat
Muscle
c
Food intake /g
Time/week
GFP+HFD
Zfyve28+HFD
d
Zfyve28
Actb
(kDa)
e
Body weight /g
P=0.852
f
Blood glucose (mmol/L)
Time (min)
P=0.005
P=0.0003
P=0.0007
P=0.0006
g
P=2×10-6
P=0.004
P=0.0008
P=0.020
P=0.003
h
i
p-Insr
Insr
p-Irs1
Irs1
p-Akt
Akt
p-Erk
Erk
j
p-Insr/Insr ratio
P=0.011
k
p-Irs1/Irs1 ratio
P=0.003
l
p-Akt/Akt ratio
P=0.041
m
p-Erk/Erk ratio
P=0.006
n
Liver weight /g
P=0.094
o
Liver/body weight (g/g)
P=0.017
p
Heart weight /g
P=0.182
q
Heart/body weight (mg/g)
P=0.157
r
Serum TG (mmol/L)
P=0.034
s
Serum T-CHO (mmol/L)
P=0.045
t
Liver TG (umol/g tissue)
P=0.038
u
Liver T-CHO (umol/g tissue)
P=0.002
v
Heart TG (umol/g tissue)
P=0.038
w
Heart T-CHO (umol/g tissue)
P=0.869
x
y
Relative blood perfusion of heart
P=0.031
z
Blood pressure (mmHg)
P=0.165
P=0.005
SBP
DBP

Supplementary Fig. 6 Liver-specific Zfyve28 overexpression markedly impaired insulin sensitivity and led to worse indicators associated with insulin resistance in mice.

a Schematic of the construction of *Zfyve28* liver-specific overexpression (LOE) mice. **b,d** qPCR (b, n=5 biologically independent samples per group) and western blotting results (d, representative results of three independent parallel experiments) showed specific overexpression of Zfyve28 in liver tissue. **c** Control mice and LOE mice showed no difference in total food intake when fed a HFD; n=5 per group. **e** Body weight of LOE mice and control mice; n=5 biologically independent mice per group. **f-g** GTT and ITT results showed that *Zfyve28* liver-specific overexpression impaired glucose tolerance (f) and insulin sensitivity (g); n=5 biologically independent mice per group. **h** Representative gross images and HE staining results of livers from control mice and LOE mice. Scale bar, 100 μm. **i-m** Western blot analysis (i) of hepatic protein levels in control mice and LOE mice. The quantification results (j-m) are shown; n=3 biologically independent samples per group. **n-q** Liver weight (n), liver/body weight ratio (o), heart weight (p), and heart/body weight ratio (q) of control mice and LOE mice; n=5 biologically independent samples per group. **r-w** The levels of serum TG (r), serum T-CHO (s), liver TG (t), liver T-CHO (u), heart TG (v) and heart T-CHO (w) in control mice and LOE mice; n=5 biologically independent samples per group. **x-y** A reduction in microcirculatory blood flow in the hearts of LOE mice was confirmed by laser Doppler blood flow measurements. Representative images (x) and quantification results of relative blood flow in five independent parallel experiments (y) are presented. Scale bar, 2 mm. **z** The SBP and DBP of control mice and LOE mice; n=5 biologically independent mice per group. LOE, liver-specific overexpression; SBP, systolic blood pressure; DBP, diastolic blood pressure; TG, triglyceride; T-CHO, total cholesterol. Data are shown as means ± SD, and *P* values are determined by unpaired two-tailed Student's *t*-test (b, e, j-w, y-z) or two-way ANOVA with Fisher's LSD post hoc multiple comparisons test (f-g). The exact *P* values are shown in the figure. Source data are provided as a Source Data file.

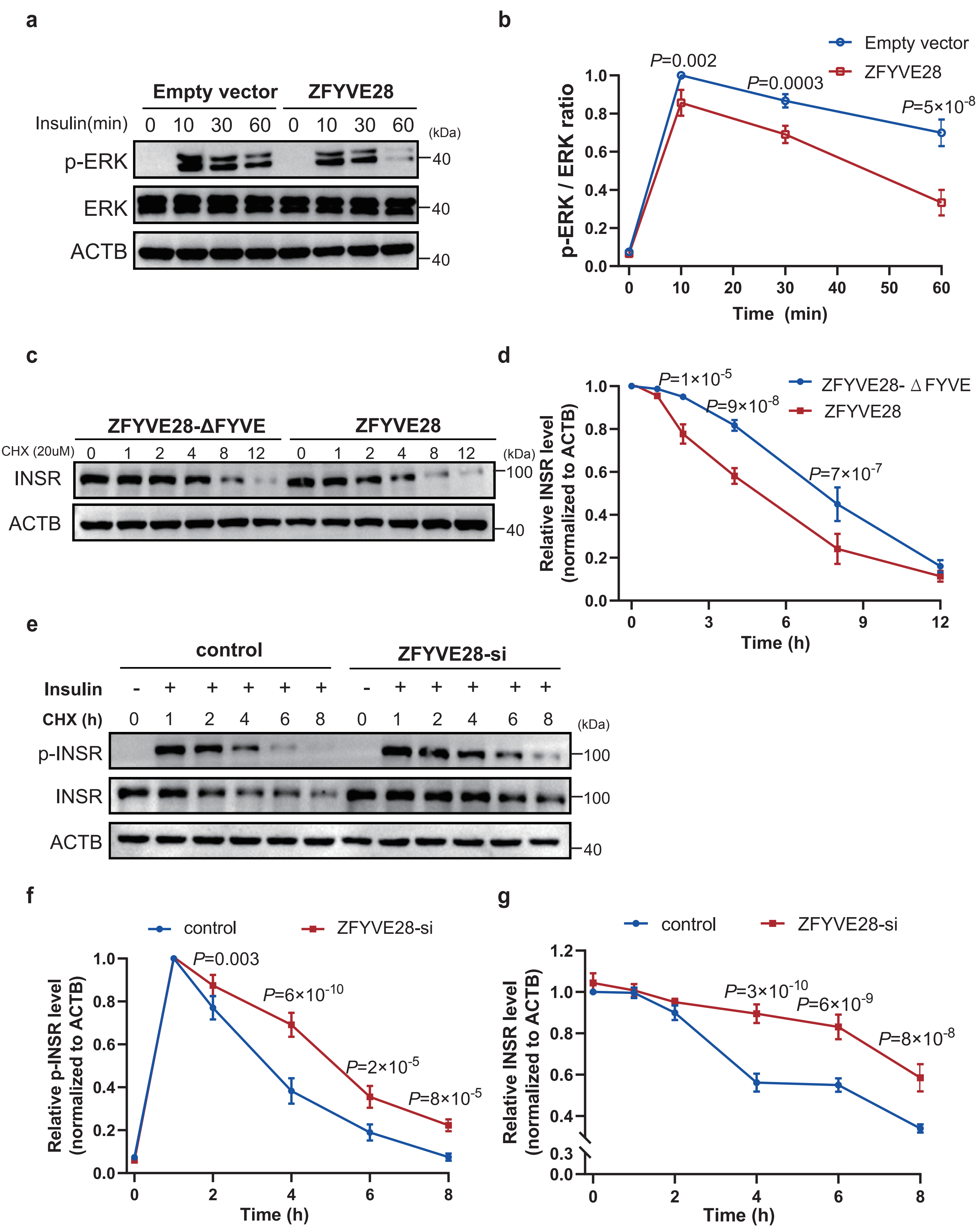
a
Empty vector
ZFYVE28
Insulin(min) 0 10 30 60 0 10 30 60
(kDa)
p-ERK
ERK
ACTB
40
40
40
b
Empty vector
ZFYVE28
P=0.002
P=0.0003
P=5×10-8
p-ERK / ERK ratio
Time (min)
c
ZFYVE28-ΔFYVE
ZFYVE28
CHX (20uM) 0 1 2 4 8 12 0 1 2 4 8 12
(kDa)
INSR
ACTB
100
40
d
ZFYVE28- Δ FYVE
ZFYVE28
P=1×10-5
P=9×10-8
P=7×10-7
Relative INSR level (normalized to ACTB)
Time (h)
e
control
ZFYVE28-si
Insulin - + + + + + - + + + + +
CHX (h) 0 1 2 4 6 8 0 1 2 4 6 8
(kDa)
p-INSR
INSR
ACTB
100
100
40
f
control
ZFYVE28-si
P=0.003
P=6×10-10
P=2×10-5
P=8×10-5
Relative p-INSR level (normalized to ACTB)
Time (h)
g
control
ZFYVE28-si
P=3×10-10
P=6×10-9
P=8×10-8
Relative INSR level (normalized to ACTB)
Time (h)

Supplementary Fig. 7 ZFYVE28 regulated phosphorylated insulin receptor degradation.

a-b Representative western blot results of p-ERK and ERK levels (a) in cells overexpressing ZFYVE28; the quantitative analysis of the p-ERK/ERK ratio (b) at different time points after insulin stimulation at 10^{-6} M of three independent experiments are shown. **c-d** Representative western blot images of INSR levels (c) in cells overexpressing ZFYVE28 and ZFYVE28-ΔFYVE with CHX (20 μM) treatment for various times, as well as the quantitative analysis results of three independent experiments (d). **e-g** Representative western blot results of p-INSR and INSR levels (e) in ZFYVE28 knockdown cells treated with insulin and CHX for various times, as well as the quantitative analysis results of three independent experiments (f-g). Data are shown as means ± SD, and *P* values are determined by two-way ANOVA with Fisher's LSD post hoc multiple comparisons test (b, d, f-g). The exact *P* values are shown in the figure. Source data are provided as a Source Data file.

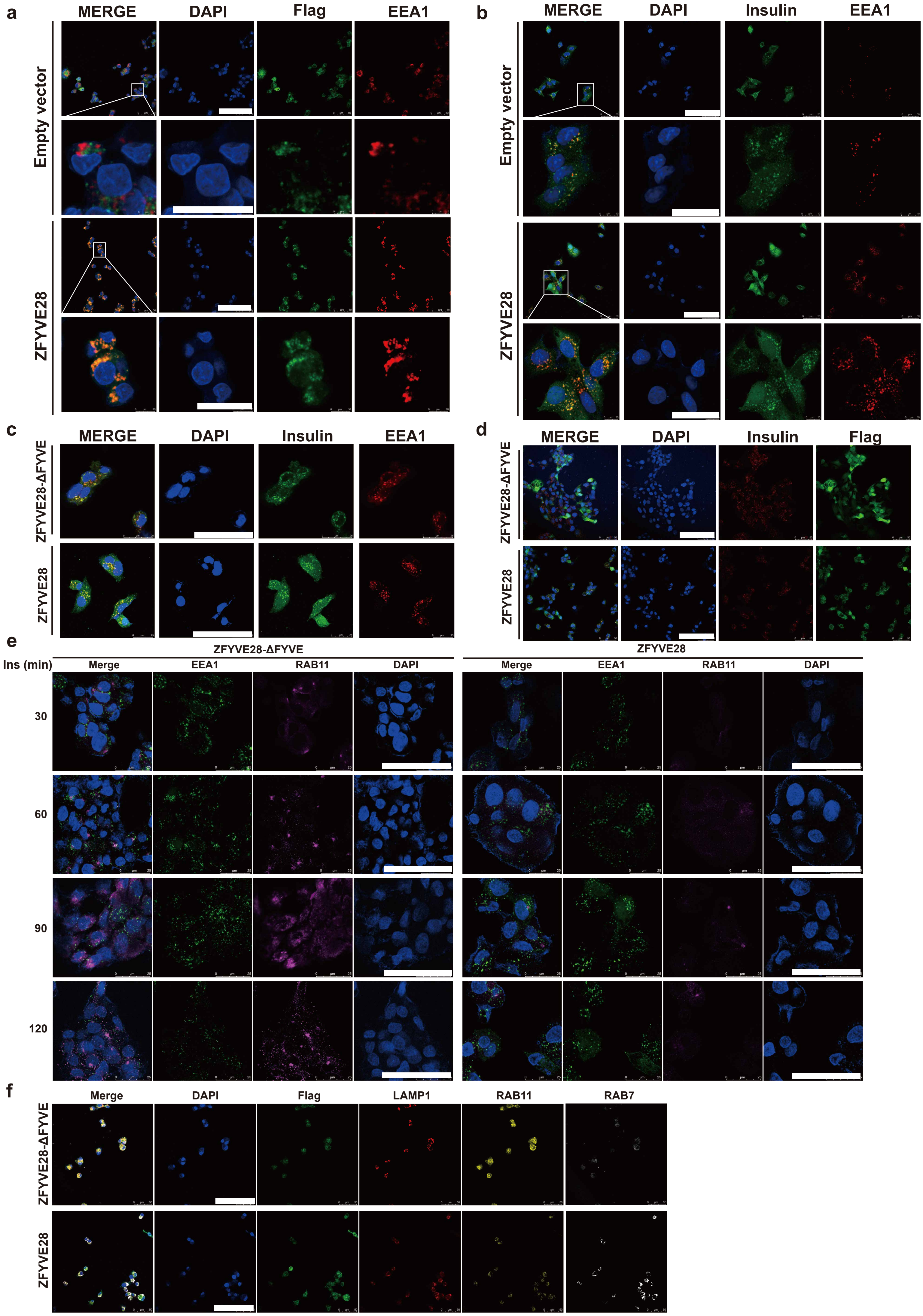
a
MERGE
DAPI
Flag
EEA1
Empty vector
ZFYVE28
b
MERGE
DAPI
Insulin
EEA1
Empty vector
ZFYVE28
c
MERGE
DAPI
Insulin
EEA1
ZFYVE28-ΔFYVE
ZFYVE28
d
MERGE
DAPI
Insulin
Flag
ZFYVE28-ΔFYVE
ZFYVE28
e
ZFYVE28-ΔFYVE
ZFYVE28
Ins (min)
Merge
EEA1
RAB11
DAPI
30
60
90
120
f
Merge
DAPI
Flag
LAMP1
RAB11
RAB7
ZFYVE28-ΔFYVE
ZFYVE28

Supplementary Fig. 8 ZFYVE28 inhibited the conversion from early endosomes to recycling endosomes.

a HepG2 cells stably overexpressed empty vector or ZFYVE28, and compared to empty vector, ZFYVE28 showed colocalization with early endosomes (EEA1-labeled endosomes). Confocal images of representative cells are shown; the magnified view of the indicated areas in the pane is also depicted (inset). Independent experiments were repeated three times in parallel. Scale bar, 100 μm, 25 μm (inset). **b** HepG2 cells overexpressing ZFYVE28 showed extensive colocalization of insulin and early endosomes. Confocal images of representative cells are shown; the magnified view of the indicated areas in the pane is also depicted (inset). Independent experiments were repeated three times in parallel. Scale bar, 100 μm, 25 μm (inset). **c** In contrast to ZFYVE28-ΔFYVE, ZFYVE28 promoted the extensive colocalization of insulin with early endosomes. Confocal images of representative cells are shown. Independent experiments were repeated three times in parallel. Scale bar, 50 μm. **d** While ZFYVE28-ΔFYVE was distributed dispersively in HepG2 cells, ZFYVE28 was more aggregated and colocalized with the insulin endocytosed into the cell. Confocal images of representative cells are shown. Independent experiments were repeated three times in parallel. Scale bar, 100 μm. **e** HepG2 cells were stimulated with insulin. Then, the cells were fixed and stained after 30, 60, 90, and 120 minutes. ZFYVE28 overexpression inhibited the generation of recycling endosomes (RAB11-labeled endosomes), whereas the recycling endosomes increased with the deletion of the FYVE domain. Confocal images of representative cells at different time points are shown. Independent experiments were repeated three times in parallel. Scale bar, 50 μm. **f** After 90 minutes of insulin stimulation at 10^{-6} M, HepG2 cells were subjected to multiplex immunofluorescence staining using Opal™ 7 color kits (Akoya Biosciences). Representative confocal images are shown. Independent experiments were repeated three times in parallel. Scale bar, 100 μm.

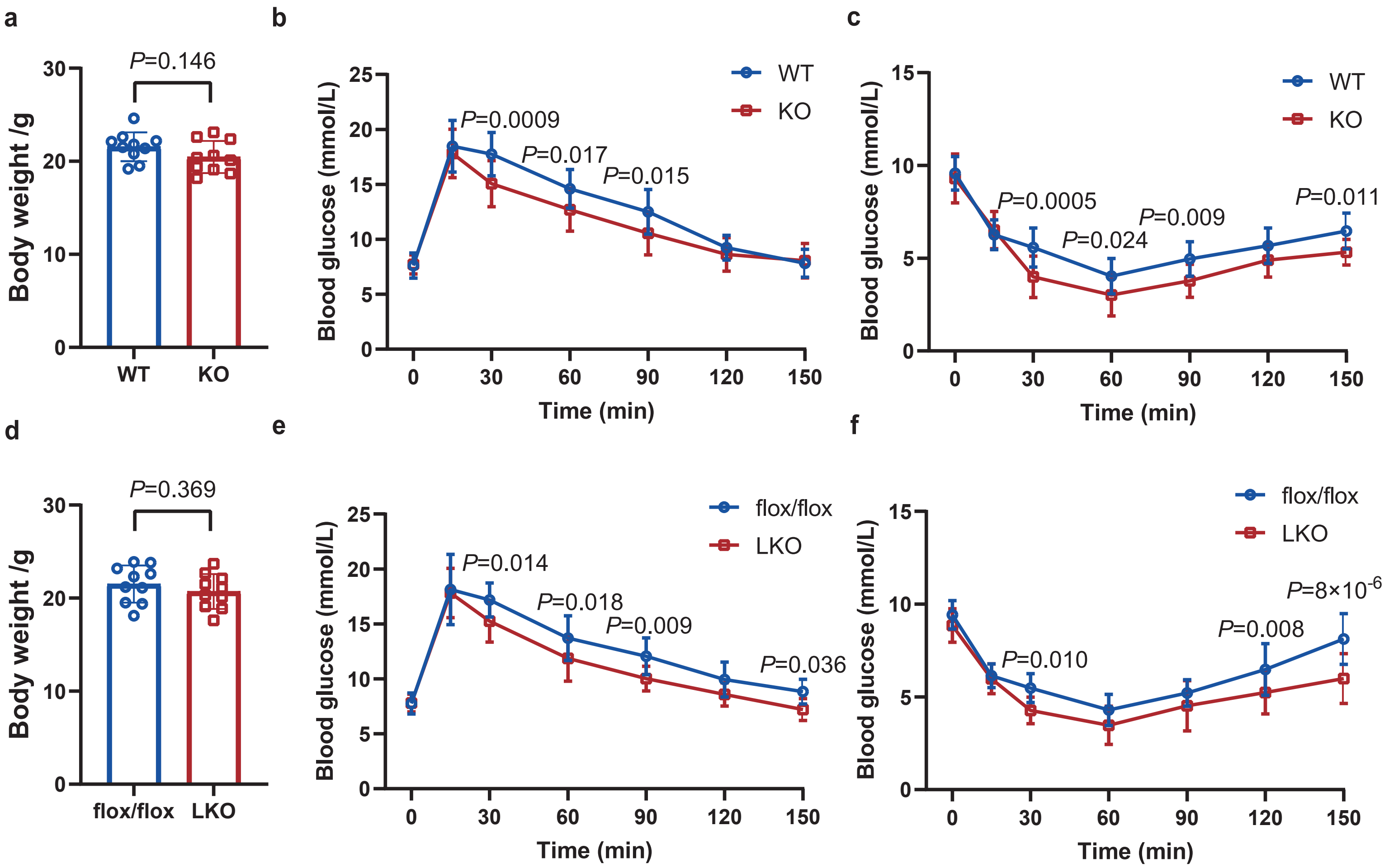

Supplementary Fig. 9 ND-fed KO and LKO mice showed better insulin sensitivity

a, d Body weight of 8-week-old control, KO and LKO mice fed a ND; n=10 biologically independent mice per group. **b-c, e-f** GTT and ITT showed better glucose tolerance and insulin sensitivity in ND-fed KO (b-c) and LKO mice (e-f) than in control mice; n=10 biologically independent mice per group. ND, normal diet. Data are shown as means ± SD, and *P* values are determined by unpaired two-tailed Student's *t*-test (a, d) or two-way ANOVA with Fisher's LSD post hoc multiple comparisons test (b-c, e-f). The exact *P* values are shown in the figure. Source data are provided as a Source Data file.

Supplementary Table 1. Baseline characteristics of study participants

Characteristics	Control	Obesity	MetS
Number	100	100	100
Men, n	50	50	50
Age, y	58.8 (4.3)	59.2 (6.0)	59.3 (5.2)
SBP, mmHg	106.2 (7.4)	109.2 (6.8) $^{P=0.003}$	138.2 (4.2) $^{P=4.09E-92}$
DBP, mmHg	69.5 (3.4)	70.4 (4.0)	83.2 (5.1) $^{P=4.78E-56}$
BMI, kg/m^2	22.1(0.9)	28.2(0.4) $^{P=1.5E-131}$	28.4(1.4) $^{P=1.32E-92}$
Waist circumference, cm	84.9 (1.8)	100.9 (2.2) $^{P=8.9E-124}$	101.8 (3.1) $^{P=1.4E-109}$
Glucose, mmol/L	5.2(0.5)	5.4(0.6) $^{P=0.011}$	7.4(1.2) $^{P=2.5E-40}$
TC, mmol/L	3.6 (1.1)	3.8 (5.7)	5.4 (2.7) $^{P=3.7E-9}$
TG, mmol/L	1.1 (2.0)	1.7 (4.8)	1.9 (2.3) $^{P=0.009}$
HDL-C, mmol/L	1.5 (0.3)	1.3 (0.4) $^{P=8.9E-5}$	1.2 (0.6) $^{P=1.3E-5}$
LDL-C, mmol/L	2.8 (0.8)	3.8 (1.0) $^{P=3.3E-13}$	3.9 (1.2) $^{P=9.8E-13}$
Smoking, n	19	16	25
Drinking, n	18	17	18
DM	0	0	100 $^{P=2.1E-45}$
CHD	0	2	26 $^{P=4.6E-8}$

Age, SBP, DBP, BMI, glucose, TC, TG, HDL-C and LDL-C values are given as means (SD) and analyzed by unpaired two-tailed Student's *t* test. Smoking, drinking, DM and CHD were analyzed by the two-sided Chi-square test. $P < 0.05$ was considered statistically significant. BMI, body mass index; CHD, coronary heart disease; DM, diabetes mellitus; TC, total cholesterol; TG, triglycerides; HDL-C, high-density lipoprotein cholesterol; LDL-C, low-density lipoprotein cholesterol. SBP, systolic blood pressure; DBP, diastolic blood pressure. Smoking and drinking status counts only current smokers and drinkers, excluding those who have quit.

Supplementary Table 2. Characteristics of participants in whom Affymetrix was performed

Characteristics	Control	Obesity	MetS
Men, n	13	13	13
Age, y	57.5 (5.0)	55.8 (5.4)	58.9 (5.7)
SBP, mmHg	106.7(7.1)	108.9 (8.3)	138.4 (12.3) $^{P=2.8E-08}$
DBP, mmHg	71.0 (5.2)	70.8 (6.6)	88.2 (13.6) $^{P=0.0003}$
BMI, kg/m^2	21.9 (0.6)	28.0 (0.7) $^{P=3.2E-18}$	29.3 (2.7) $^{P=9.9E-10}$
Waist circumference, cm	84.2 (2.3)	100.5 (3.8) $^{P=1.6E-12}$	100.7 (8.8) $^{P=9.2E-07}$
Glucose, mmol/L	5.3(0.3)	5.6 (0.5)	7.5 (1.5) $^{P=2.6E-05}$
Insulin, uIU/ml	10.8(1.9)	15.9(2.5) $^{P=4.9E-06}$	23.5(3.2) $^{P=7.4E-12}$
TC, mmol/L	4.9 (0.7)	5.4 (0.9)	5.9 (0.9) $^{P=0.004}$
TG, mmol/L	1.0 (0.6)	1.4 (3.4)	1.4 (2.4)
HDL-C, mmol/L	1.3(0.2)	1.4(0.4)	1.1 (0.4)
LDL-C, mmol/L	2.9(0.5)	3.3(0.7)	3.5(1.7)
Smoking, n	1	2	6 $^{P=0.027}$
Drinking, n	1	0	2
DM	0	0	13 $^{P=3.4E-7}$
CHD	0	0	3

Age, SBP, DBP, BMI, glucose, insulin, TC, TG, HDL-C and LDL-C values are given as means (SD) and analyzed by unpaired two-tailed Student's *t* test. Smoking, drinking, DM and CHD were analyzed by the two-sided Chi-square test. $P < 0.05$ was considered statistically significant. BMI, body mass index; CHD, coronary heart disease; DM, diabetes mellitus; TC, total cholesterol; TG, triglycerides; HDL-C, high-density lipoprotein cholesterol; LDL-C, low-density lipoprotein cholesterol. Smoking and drinking status counts only current smokers and drinkers, excluding those who have quit.

Supplementary Table 3. Primers for RT-qPCR

Genes	F/R	Sequences
ZFYVE28	F	5’-TGCGGGACCTGAACACCTA-3’
	R	5’-CATGGCCGAGACGTAGCTG-3’
HES1	F	5’-TCAACACGACACCGGATAAAC-3’
	R	5’-GCCGCGAGCTATCTTTCTTCA-3’
HEY1	F	5’-GTTCGGCTCTAGGTTCCATGT-3’
	R	5’-CGTCGGCGCTTCTCAATTATTC-3’
ACTB	F	5’-CATGTACGTTGCTATCCAGGC-3’
	R	5’-CTCCTTAATGTCACGCACGAT-3’
Zfyve28	F	5’-CATCATGAATCGGGAGCTGGA-3’
	R	5’-GTGTTGAGGTCTCGGAGTGC-3’
Actb	F	5’-GTGACGTTGACATCCGTAAAGA-3’
	R	5’-GCCGGACTCATCGTACTCC-3’

Supplementary Table 4. Antibodies and dilutions

Antibodies	Brands	Dilutions
ZFYVE28	Biorbyt, orb28487	1:500; 1:1000
ACTB (HRP-conjugated)	Proteintech, HRP-60008	1:5000
INSR	CST, 3025S	1:1000
p-INSR	CST, 3026S	1:1000
AKT	CST, 4685S	1:1000
p-AKT	CST, 13038S	1:1000
ERK	CST, 4695S	1:1000
p-ERK	CST, 4370S	1:1000
NOTCH1	CST, 3608S	1:1000
NICD	CST, 4147S	1:1000
RAS-GTP	CST, 8821S	1:200
IRS1	CST, 95816S	1:1000
p-IRS1	CST, 2388S	1:1000
HRP-linked anti-rabbit IgG	CST, 7074S	1:5000
Lamp1	Proteintech, 67300-1-Ig	1:100
Insulin	CST, 3014S	1:100
flag	CST, 14793S, 8146S	1:400
Eea1	CST, 48453S	1:100
Rab7	CST, 9367S, 95746S	1:100, 1:1000
Rab11	CST, 5589S; Proteintech, 15903-1	1:100, 1:1000
Goat anti-Mouse IgG, Alexa Fluor™ 488	Invitrogen, A-11029	1:500
Goat anti-Mouse IgG, Alexa Fluor™ 594	Invitrogen, A-11005	1:500
Goat anti-Rabbit IgG, Alexa Fluor™ 488	Invitrogen, A-11034	1:500
Goat anti-Rabbit IgG, Alexa Fluor™ 594	Invitrogen, A-11012	1:500
