# Supplementary material for: ZFYVE28 mediates insulin resistance by promoting phosphorylated insulin receptor degradation via increasing late endosomes production
Source: Nat Commun. 2023 Oct 26;14:6833. doi: 10.1038/s41467-023-42657-w (PMC10603069; doi:10.1038/s41467-023-42657-w)
Supplement: Supplementary file 3 — Description of Additional Supplementary Files [file 41467_2023_42657_MOESM3_ESM.pdf]

Description of Additional Supplementary Files

Title: **Supplementary Data 1**

Description: Source data for Figure 1b, showing all differentially expressed genes between insulin-sensitive obese patients and healthy controls (13 males vs. 13 males), and ZFYVE28 expression is downregulated in insulin-sensitive obese patients.

Title: **Supplementary Data 2**

Description: Source data for Figure 1d, showing all differentially expressed genes between insulin-resistant MetS (metabolic syndrome) patients and healthy controls (13 males vs. 13 males), and ZFYVE28 expression is upregulated in insulin resistant patients.
