# Supplementary material for: ZFYVE28 mediates insulin resistance by promoting phosphorylated insulin receptor degradation via increasing late endosomes production
Source: Nat Commun. 2023 Oct 26;14:6833. doi: 10.1038/s41467-023-42657-w (PMC10603069; doi:10.1038/s41467-023-42657-w)
Supplement: Supplementary file 6 — Reporting Summary [file 41467_2023_42657_MOESM6_ESM.pdf]

nature portfolio

Corresponding author(s): Yibo Wang

Last updated by author(s): Aug 29, 2023

Reporting Summary

Nature Portfolio wishes to improve the reproducibility of the work that we publish. This form provides structure for consistency and transparency in reporting. For further information on Nature Portfolio policies, see our Editorial Policies and the Editorial Policy Checklist.

Statistics

For all statistical analyses, confirm that the following items are present in the figure legend, table legend, main text, or Methods section.

n/a	Confirmed	
☐	☒	The exact sample size (*n*) for each experimental group/condition, given as a discrete number and unit of measurement
☐	☒	A statement on whether measurements were taken from distinct samples or whether the same sample was measured repeatedly
☐	☒	The statistical test(s) used AND whether they are one- or two-sided<br>*Only common tests should be described solely by name; describe more complex techniques in the Methods section.*
☐	☒	A description of all covariates tested
☐	☒	A description of any assumptions or corrections, such as tests of normality and adjustment for multiple comparisons
☐	☒	A full description of the statistical parameters including central tendency (e.g. means) or other basic estimates (e.g. regression coefficient) AND variation (e.g. standard deviation) or associated estimates of uncertainty (e.g. confidence intervals)
☐	☒	For null hypothesis testing, the test statistic (e.g. *F*, *t*, *r*) with confidence intervals, effect sizes, degrees of freedom and *P* value noted<br>*Give P values as exact values whenever suitable.*
☒	☐	For Bayesian analysis, information on the choice of priors and Markov chain Monte Carlo settings
☒	☐	For hierarchical and complex designs, identification of the appropriate level for tests and full reporting of outcomes
☒	☐	Estimates of effect sizes (e.g. Cohen's *d*, Pearson's *r*), indicating how they were calculated

Our web collection on statistics for biologists contains articles on many of the points above.

Software and code

Policy information about availability of computer code

Data collection	The following tools were used for data collection:<br>-Spectrophotometer (Nanodrop 2000c, Thermo Fisher)<br>-Prism 7500 sequence-detection system (ABI, Rockford, IL)<br>-Affymetrix GeneChip® Human Gene 2.0 ST Array (Affymetrix Inc.)<br>-Leica DM4000B with a digital camera (Leica, NY)<br>-Leica SP8 Laser Confocal Microscope (Germany)<br>-MRBP blood pressure system (IITC Life Science)<br>-Laser Doppler flowmetry (PeriCam PSI System, T402-PT)<br>-Accu-Chek Active Blood Glucose Meter (Roche)<br>-Microplate reader (Infinite-M200)<br>-Chemiluminescence system (Tanon 5800 Multi, China)<br>-JASPAR online tools (https://jaspar.genereg.net)
Data analysis	The following software were used for data analysis:<br>-Image J software (version 2.0.0) was used for quantitative analysis.<br>-GraphPad Prism (version 9.0) and SPSS (version 26.0) were used for statistical analysis.<br>-R (version 4.0.4), R packages fgsea (version 1.26.0) and clusterProfiler (version 4.8.2) were used for gene set enrichment analysis.<br>All the replicate experiments (including cell and mouse-based experiments) are biological replicates, which are repeated at least three times. Data are presented as the mean ± SD and individual data point is plotted. The normality of the data was tested using the Shapiro–Wilk normality test. The differences between two groups were evaluated using unpaired Student’s t tests, and multiple group comparisons were

conducted by one-way ANOVA followed by Tukey's post hoc tests or two-way ANOVA with Fisher's LSD post hoc multiple comparisons test. A P value < 0.05 was considered statistically significant. Statistical analyses were performed in GraphPad Prism 9.0 and SPSS.26.0. The images were created by Adobe Illustrator 2020 and Adobe Photoshop 2020 software.

For manuscripts utilizing custom algorithms or software that are central to the research but not yet described in published literature, software must be made available to editors and reviewers. We strongly encourage code deposition in a community repository (e.g. GitHub). See the Nature Portfolio guidelines for submitting code & software for further information.

Data

Policy information about availability of data

All manuscripts must include a data availability statement. This statement should provide the following information, where applicable:

- Accession codes, unique identifiers, or web links for publicly available datasets
- A description of any restrictions on data availability
- For clinical datasets or third party data, please ensure that the statement adheres to our policy

The authors confirm that all the data supporting the findings of this study are available in the paper and its supplementary information and Source Data file. Additional information and derived data supporting the findings of this study are available upon reasonable request and subject to a data use agreement from the corresponding author (Y.W.). Source data are provided with this paper.

Research involving human participants, their data, or biological material

Policy information about studies with human participants or human data. See also policy information about sex, gender (identity/presentation), and sexual orientation and race, ethnicity and racism.

Reporting on sex and gender	The study population was from Rizhao City in the northern region of China from 2009 to 2010, including 100 patients with obesity, 100 patients with MetS and another 100 matched normal controls, part of which had been described previously (PMID: 34615377, 34397273, 25280487). Half were males and half were females. The Information of participants is presented in detail in the manuscript and supplementary materials.
Reporting on race, ethnicity, or other socially relevant groupings	The study population was from Rizhao City in the northern region of China from 2009 to 2010, including 100 patients with obesity, 100 patients with MetS and another 100 matched normal controls, part of which had been described previously (PMID: 34615377, 34397273, 25280487). All participants are Chinese Han people.
Population characteristics	The following strict inclusion criteria were used for obese patients, MetS patients and controls: 1) Chinese Han people; 2) aged 50-77 years; and 3) subjects were excluded when they had any known diseases including thyroid disease, hematological diseases, peptic ulcers, liver or kidney dysfunctions, infections, autoimmune diseases, or tumors. According to Chinese body mass index (BMI) standards, underweight, normal weight, and overweight/obesity were defined as BMI <18.5, 18.5–23.9, and ≥24.0 kg/m2, respectively. The controls had normal BMI (18.5–23.9), while the patients with obesity had BMI≥28.0 or BMI>27.0 and waist circumference >101 cm. All obese patients were nondiabetic and had normal insulin sensitivity. The more strict criteria for MetS patients were BMI≥ 28.0 or BMI>27.0 and waist circumference >101 cm, plus three or more of the following: 1) elevated triglycerides (drug treatment for elevated triglycerides was an alternate indicator) ≥1.7 mmol/L; 2) reduced HDL-C (drug treatment for reduced HDL-C was an alternate indicator) < 1.0 mmol/L; 3) elevated blood pressure (antihypertensive drug treatment in a patient with a history of hypertension was an alternate indicator) with current or previous SBP ≥160 mmHg and DBP ≥100 mmHg; and 4) elevated fasting glucose (drug treatment of elevated glucose was an alternate indicator) ≥ 6.1 mmol/L. Moreover, these 100 MetS patients were all diabetic. The basic characteristics are shown in Supplementary Table 1. The relevant information is described in detail in the manuscript and supplementary files.
Recruitment	All participants were recruited from those people in the Rizhao Port community who underwent routine health examinations at Rizhao Port Hospital between 2009 and 2010. According to Chinese body mass index (BMI) standards (Zhou et al., 2002; Lu et al., 2009), the controls had normal BMI (18.5–23.9), while the patients with obesity had BMI≥28.0 or BMI>27.0 and waist circumference >101 cm. The diagnostic criteria for metabolic syndrome referred to the American Heart Association definition of metabolic syndrome (Alberti et al., 2009). All participants gave their signed informed consent to the study. There was no self-selection bias or any other bias during the recruitment of individuals in this study.
Ethics oversight	The study was reviewed and approved by the ethics committees of Fuwai Hospital and Rizhao Port Hospital.

Note that full information on the approval of the study protocol must also be provided in the manuscript.

Field-specific reporting

Please select the one below that is the best fit for your research. If you are not sure, read the appropriate sections before making your selection.

☒ Life sciences ☐ Behavioural & social sciences ☐ Ecological, evolutionary & environmental sciences

For a reference copy of the document with all sections, see nature.com/documents/nr-reporting-summary-flat.pdf

Life sciences study design

All studies must disclose on these points even when the disclosure is negative.

Sample size	The N number for all experiments, including animal experiments, in vitro experiments and clinical sample studies were listed in the figure

	legends or in Supplementary Table 1-2. The sample size was chosen based on our prior studies (PMID: 34615377, 34397273)and other previous papers with similar experiments (PMID: 28924165, 25628421, 36158197), which showed sufficient statistical power for in vitro experiments and animal experiments.
Data exclusions	No samples or animals were excluded from analyses.
Replication	All animal experiments were repeated at least twice and in vitro experiments were repeated at least three times. All results are reproducible and representative data were showed in the figures or supplementary files.
Randomization	Animals were allocated to their respective group at birth by a blinded investigator. For other experiments, including cell experiments, before performing the corresponding treatment, samples were randomly assigned to control and experimental groups by an investigator blinded to subsequent experimental information. The standard laboratory procedures were strictly followed to keeping the experimental environment and facilities consistent and performed under the same conditions.
Blinding	Investigators were blinded to group allocation during data collection, image quantification and data analysis.

Reporting for specific materials, systems and methods

We require information from authors about some types of materials, experimental systems and methods used in many studies. Here, indicate whether each material, system or method listed is relevant to your study. If you are not sure if a list item applies to your research, read the appropriate section before selecting a response.

Materials & experimental systems

n/a	Involved in the study
☐	☒ Antibodies
☐	☒ Eukaryotic cell lines
☒	☐ Palaeontology and archaeology
☐	☒ Animals and other organisms
☒	☐ Clinical data
☒	☐ Dual use research of concern
☒	☐ Plants

Methods

n/a	Involved in the study
☒	☐ ChIP-seq
☒	☐ Flow cytometry
☒	☐ MRI-based neuroimaging

Antibodies

Antibodies used	ZFYVE28 (Biorbyt, #orb28487), HRP-conjugated ACTB (Proteintech, #HRP-60008), INSR (CST, #3025S), p-INSR (CST, #3026S), AKT (CST, #4685S), p-AKT (CST, #13038S), ERK (CST, #4695S), p-ERK (CST, #4370S), NOTCH1 (CST, #3608S), NICD (CST, #4147S), HRP-linked anti-rabbit IgG (CST, #7074S), Lamp1 (Proteintech, #67300-1-Ig), Insulin (CST, #3014S), IRS1 (CST, #95816S), p-IRS1 (CST, #2388S), Flag (CST, #14793S, #8146S), Eea1 (CST, #48453S), Rab7 (CST, #9367S, #95746S), Rab11 (CST, #5589S;Proteintech, #15903-1-AP), RAS-GTP (CST, #8821S), Alexa Fluor™ 488 Goat anti-Mouse IgG (Invitrogen, #A-11029), Alexa Fluor™ 594 Goat anti-Mouse IgG (Invitrogen, # A-11005), Alexa Fluor™ 488 Goat anti-Rabbit IgG (Invitrogen, #A-11034), Alexa Fluor™ 594 Goat anti-Rabbit IgG (Invitrogen, #A-11012)
Validation	The antibodies including ZFYVE28, ACTB, INSR, p-INSR, AKT, p-AKT, ERK, p-ERK, NOTCH1, NICD, IRS1, p-IRS1, RAS-GTP, Rab7 and Rab11 were validated for the western blotting of both human and mouse samples on the websites of the associated companies (https://biorbyt.com.cn/, https://www.ptgcn.com/, https://www.cellsignal.cn/). Rab7 and Rab11 antibodies were validated for the immunohistochemical staining of human samples on the websites of the companies (https://www.ptgcn.com/, https://www.cellsignal.cn/). The antibodies including Lamp1, Insulin, Flag, Eea1, Rab7 and Rab11 were validated for the immunofluorescence staining of human samples on the websites of the associated companies (https://www.ptgcn.com/, https://www.cellsignal.cn/).

Eukaryotic cell lines

Policy information about cell lines and Sex and Gender in Research

Cell line source(s)	HepG2 cells (ATCC, HB-8065), HEK293T cells (ATCC, CRL-3216) and HeLa cells (ATCC, CCL-2) were all obtained from ATCC.
Authentication	Authentication of all the cell lines were performed by a Human STR Profiling Cell Authentication Service (ATCC).
Mycoplasma contamination	Cells tested negative for mycoplasma contamination.
Commonly misidentified lines (See ICLAC register)	No misidentified lines were used in the study.

Animals and other research organisms

Policy information about studies involving animals; ARRIVE guidelines recommended for reporting animal research, and Sex and Gender in Research

Laboratory animals	Both Zfyve28 global knockout (KO) mice and liver-specific knockout (LKO) mice (C57BL/6J background) were constructed at Cyagen Biosciences (Suzhou, China). The mice were housed under a 12-hour light/dark cycle at a temperature of 23 ± 1 °C and relative humidity of 50%-60%, with free access to water. Unless mentioned otherwise, 6-week-old mice were used for experiments.
Wild animals	The study did not involve any wild animal.
Reporting on sex	The mice used in the study were all male due to their lower sex hormone variations, greater susceptibility to obesity and more pronounced impairment of insulin sensitivity under high-fat diet induction. Relevant study information is described detailly in the manuscript and supplementary materials.
Field-collected samples	No field-collected samples were used in the study.
Ethics oversight	All animal use and welfare adhered to the National Institutes of Health's Guide for the Care and Use of Laboratory Animals following a protocol reviewed and approved by the State Key Laboratory of Cardiovascular Disease, National Center for Cardiovascular Diseases, Fuwai Hospital (Beijing, China; permit number: 0000869). The study was reviewed and approved by the ethics committee of Fuwai Hospital (Bejing, China; No. FW-2022-0049).

Note that full information on the approval of the study protocol must also be provided in the manuscript.
